# Cluster analysis identifies a pathophysiologically distinct subpopulation with increased serum leptin levels and severe obstructive sleep apnea

**DOI:** 10.1007/s11325-020-02160-8

**Published:** 2020-09-04

**Authors:** Yutaka Kozu, Yusuke Kurosawa, Shiho Yamada, Asami Fukuda, Mari Hikichi, Hisato Hiranuma, Toshiki Akahoshi, Yasuhiro Gon

**Affiliations:** Division of Respiratory Disease, Nihon University School of Medicine, 30-1 Ohyaguchi-Kamicho, Itabashiku, Tokyo, 173-8610 Japan

**Keywords:** Obstructive sleep apnea, Phenotypes, Cluster analysis, Leptin, Blood gas analysis

## Abstract

**Purpose:**

To investigate the different pathophysiologies of obstructive sleep apnea (OSA) phenotypes using cluster analysis. Differences between leptin/adiponectin levels in the resulting OSA phenotypes were also examined.

**Methods:**

In total, 1057 OSA patients were selected, and a retrospective survey of clinical records, polysomnography results, and blood gas data was conducted. Patients were grouped into four clusters by their OSA severity, PaCO2, body mass index (BMI), and sleepiness. A *k*-means cluster analysis was performed, resulting in a division into four subpopulations. The Tukey or Games-Howell tests were used for intergroup comparisons.

**Results:**

Among the 20 clinical OSA items, four common factors (Epworth Sleepiness Scale [ESS], BMI, Apnea-Hypopnea Index [AHI], and PaCO2) were extracted by principal component analysis, and a cluster analysis was performed using the *k*-means method, resulting in four distinct phenotypes. The Clusters 1 (middle age, symptomatic severe OSA) and 4 (young, obese, symptomatic very severe OSA) exhibited high leptin levels. C-reactive protein levels were also elevated in Cluster 4, indicating a different pathophysiological background. No apparent differences between clusters were observed regarding adiponectin/leptin ratios and adiponectin levels. Classification into groups based on phenotype showed that Epworth Sleepiness Scale [ESS] score and disease severity were not correlated, suggesting that sleepiness is affected by multiple elements.

**Conclusions:**

The existence of multiple clinical phenotypes suggests that different pathophysiological backgrounds exist such as systemic inflammation and metabolic disorder. This classification may be used to determine the efficacy of continuous positive airway pressure treatment that cannot be determined by the AHI.

## Introduction

The prevalence of obstructive sleep apnea (OSA) was reported 20 years ago by Young and colleagues, affecting up to 9% of men and 4% of women [1–4]. Although there have been no major epidemiological studies in Japan, OSA prevalence rates in men in their 40s and 50s are 23.4% and 30.6%, respectively [2]. The OSA prevalence in Japan is comparable with that in Europe and the USA.

OSA substantially complicates cardiovascular diseases and they are important factors in determining the OSA prognosis. Repeated nocturnal hypoxemia is an important factor, and hypertension or diabetes mellitus is frequently involved. Thus, OSA is considered to overlap with various risk factors related to cardiovascular system disorders [3]. It is also recognized as being composed of multiple phenotypes; its diagnosis and management as well as the assessment of its severity are often based on a single indicator, the Apnea-Hypopnea Index (AHI) [5]. Taking the diverse OSA pathologies into consideration is important for a more fine-tuned OSA management.

Cytokines secreted from adipose tissue play an important role in the pathophysiology of obesity; the relationship between obesity and sleep-disordered breathing is well-known. Among adipokines, leptin and adiponectin have attracted the most attention [6]. Despite the anti-obesity effects of leptin, obese individuals have elevated serum leptin levels, and administering leptin to obese individuals only produces an extremely limited impact on obesity. Since this is the result of reduced sensitivity to leptin signaling, leptin resistance is involved in obesity [7]. Leptin does not only act in the appetite center but is also involved in the ventilatory response [8].

There have been recent efforts to stratify patients by using clinical findings as indicators to understand the disease mechanism, predict risk, estimate prognosis, and select optimal treatment [9]. Patient populations classified in this manner are called “phenotypes” and may not only reflect classification by clinical features but also pathophysiological characteristics.

Taking the different phenotypes of OSA into consideration has the potential to better understand the underlying pathophysiology and to properly manage treatment according to trends in arousal, ventilatory sensitivity, and other pathophysiological features [10]. The main advantages of numerical classification are its objectivity and the fact that a methodology for including multiple variables, assuming equal weighting, helps minimize a priori bias and extract clusters that are important for exploratory purposes.

To classify the clinical OSA phenotypes, we hypothesized that it would be possible to identify the most important factors from variables made up of multiple clinical indicators and that the subsequent cluster analysis would enable us to identify phenotypes reflective of different OSA pathophysiologies. To test this hypothesis, a *k*-means clustering algorithm was used in the present study to investigate mild-to-severe OSA diagnosed based on polysomnography (PSG) testing at our center.

Therefore, this study aimed to investigate the different pathophysiological OSA phenotypes by clustering patients with OSA according to different clinical indicators and examine the cluster differences in leptin and adiponectin levels.

## Materials and methods

Our study was approved by the Clinical Research Ethics Committee of the Nihon University Hospital (Protocol number RK-170509-07). Written informed consent was waived by the ethics committee. All protocols and practices were conducted following the World Medical Association’s Declaration of Helsinki.

### Subjects

Medical records of 3214 patients with obstructive sleep apnea among patients, who visited the Nihon University Sleep Center between April 1, 2002, and March 31, 2017, with suspected sleep apnea syndrome and were diagnosed based on the PSG results, were registered in a database. Overnight PSG was performed in 2191 patients with suspected OSA who visited Nihon University Itabashi Hospital Sleep Center; of these, 2174 were diagnosed with OSA. Of these, 1057 individuals whose clinical records, PSG data, and blood gas data were available, were finally enrolled in the present study. The following patients were excluded: OSA patients who did not agree to participate in the study, those who were aged < 20 years, and those who had not undergone PSG and respiratory function tests.

### Polysomnography

PSG was conducted by trained professional inspectors in all cases. The following items were recorded: electroencephalogram, electrooculogram, electrocardiogram, electromyogram, nose and mouth airflow, chest and abdomen movement, and peripheral capillary oxygen saturation (SpO2). Apnea was defined as airflow cessation in the nose and mouth lasting at for least 10 s. Hypopnea was defined as decreased airflow, thoracic excursion, or decreased oxygen desaturation below 4% of the previous baseline value and decreased abdominal excursion below 50%. The AHI was calculated as the apnea and hypopnea counts per hour of sleep. The diagnostic criteria for OSA were AHI ≥ 5 times/h. The severity was defined as mild for AHI 5–15 times/h, moderate for 15–30 times/h, and severe for ≥ 30 times/h. The CPAP adaptation standard in Japan is AHI ≥ 20 times/h, and most cases in this study had an AHI of 20 times/h or more.

Average and minimum SpO2 values were also calculated from the PSG data. Baseline clinical features were assessed, and blood tests were also performed.

### Blood tests

For blood counts and general biochemical testing, data were obtained from general medical records. Serum leptin and adiponectin levels were measured in all patients when residual samples were available after the blood test. Leptin and adiponectin were measured using the Human Leptin Assay Kit (catalog number #27775; IBL, Inc., Gunma, Japan) and Human Adiponectin ELISA Kit (catalog number #CY-8050; CircuLex, Inc., Nagano, Japan), respectively.

### Spirometry

Conventional spirometry was performed using a Chestak auto-spirometer (Chest Co., Tokyo, Japan). Spirometric predictions were obtained from the literature [11], and samples for the arterial blood gas analysis were taken from the radial artery in a sitting position.

The criterion for obstructive ventilatory dysfunction was defined as 70% or less per second, and the criterion for restrictive ventilatory dysfunction was 80% or less vital capacity.

### Blood gas analysis

Arterial blood samples were analyzed using an ABL3000 auto-analyzer (Radiometer Co., Tokyo, Japan).

### ESS score

Daytime sleepiness was assessed using the Epworth Sleepiness Scale (ESS) [12], a fully validated eight-item self-administered questionnaire. Eight different situations were scored on a scale of 0–3, with more than 10 out of 24 scores rated as sleepiness.

### Statistical methods

**Fig. 1:**
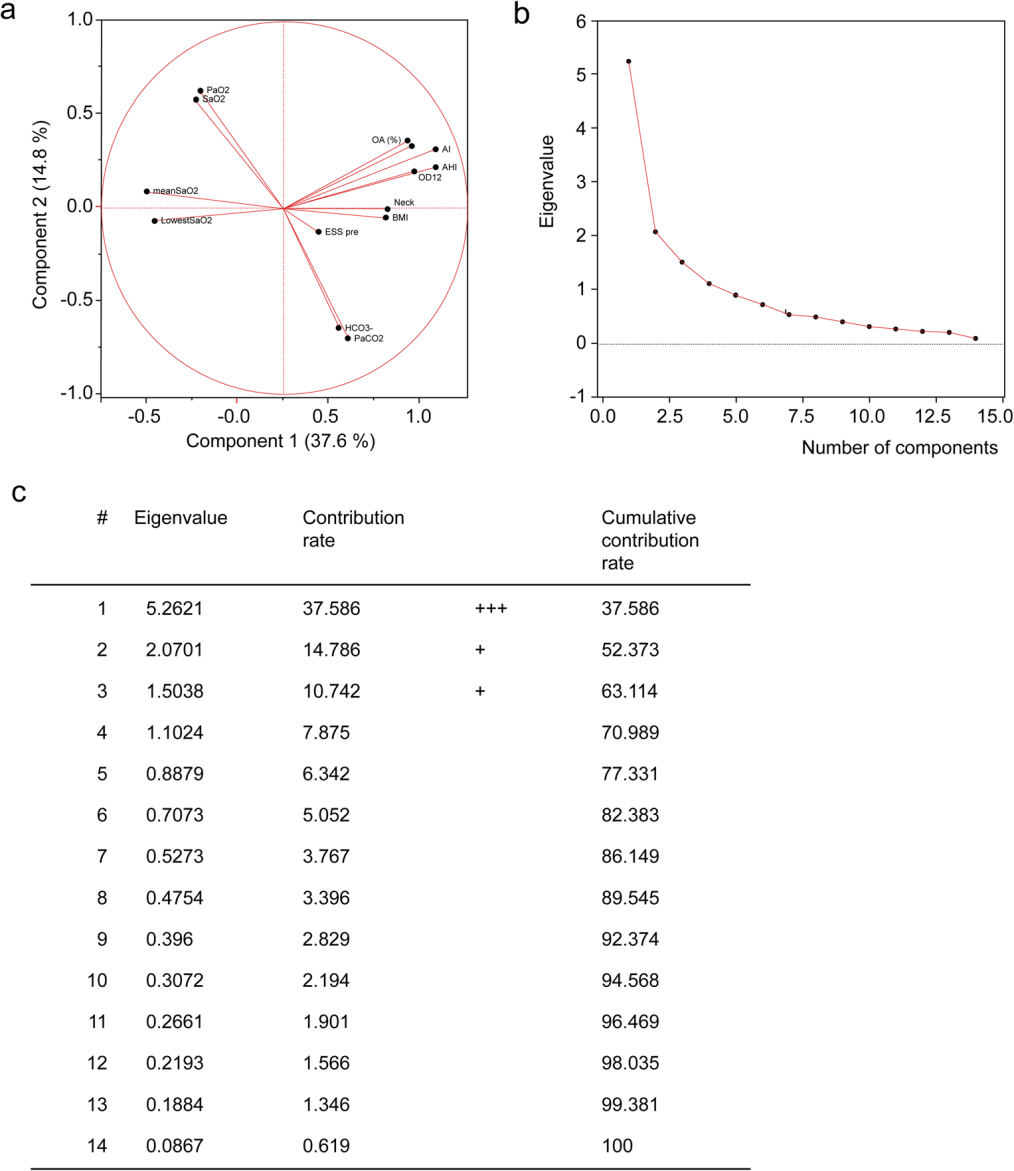
Principal component analysis

The *k*-means method was considered to be suitable considering that a relatively large number of samples were classified into four clusters in this study. The principal component analysis was used as a simple method for confirming the clustering of data (Fig. 1). The required number of subjects was determined from the independent variables while performing a multi-group comparison of each cluster, and the sample size was sufficient (*n* > 64) even when the power was set to 80%. Results are presented as mean ± standard deviation (SD). AHI, partial pressure of carbon dioxide (PaCO2), body mass index (BMI), and ESS were obtained as four higher level components from eigenvalues, contribution rates, and cumulation.

**Fig. 2:**
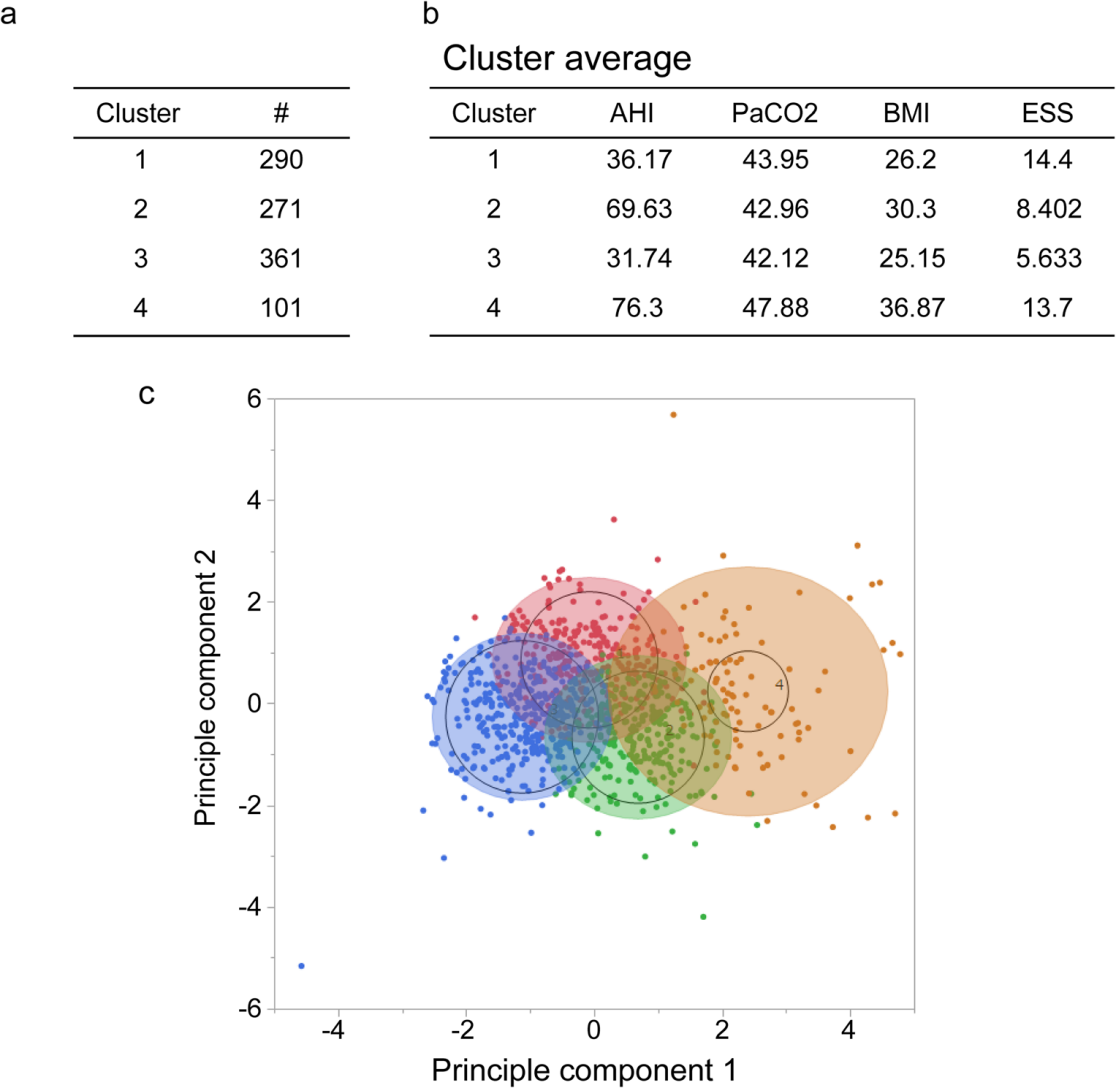
Cluster analysis (*k*-means)

The *k*-means cluster analysis was performed based on these four principal components, resulting in four subpopulations (Fig 2). The resulting data were tested for equality of variance between the clusters in each of the items. For equal variance, the one-way ANOVA with Tukey’s all column comparison test was used for intergroup comparisons. Otherwise, intergroup comparisons were performed using the nonparametric Games-Howell test. If an *F*-value was > 4, *p* values < 0.05 were considered statistically significant. (Text Explorer module of JMP Pro 13)

## Results

### Patient characteristics

Of the 1057 registered patients, 24 (3.2%) had incomplete or absent data, or chiefly presented central sleep apnea or hypopnea. These patients were excluded from the analysis. The remaining 1023 patients diagnosed with OSA had a mean age of 51.4 years, ranging from 20 to 83 years, with 88% being men. The percentage of current smokers was around 24%. The mean neck circumference was about 40 cm, which was higher than an average Japanese adult. The self-reported day time sleepiness was assessed using the ESS and the mean score was 9.7 points (Table 1).

**Table 1:** Patient characteristics

		All clusters *N* = 1023
Age	Year	51.41
Female	Number	121
Sex (female)	%	11.8
Smoker	(*n*)	249
Ex-smoker	(*n*)	262
Body mass index	(kg/m^2^)	27.97
Neck	(cm)	40.27
Waist	(cm)	95.50
Hip	(cm)	104.17
ESS score		9.65

*ESS* Epworth Sleepiness Scale

The cluster analysis identified four clusters. Compared with the Clusters 1 and 2, Cluster 3 was older and the Cluster 4 younger. There were no significant differences in male-female ratio or history of smoking between the clusters. The BMI values, as well as the neck, waist, and hip circumferences, were highest in Cluster 4. ESS scores were elevated in Clusters 1 and 4 (Table 2).

**Table 2:** Summary of the main features in the identified clusters

Patient characteristics		All clusters			Cluster 1			Cluster 2			Cluster 3			Cluster 4			
		*N* = 1023			*N* = 290			*N* = 271			*N* = 361			*N* = 101			*p* value
Age	Year	51.41	±	13.34	51.82	±	12.71	49.10	±	12.93	55.24	±	13.29	42.71	±	10.96	< 0.0001
Sex	(F/M)	121 / 902			29 / 261			29 / 242			54 / 307			9 / 92			
	Female %	11.8			10.0			10.7			15.0			8.9			
Smoker	(*n*)	249			58			67			86			38			
Ex-smoker	(*n*)	262			82			46			110			24			
BMI	(kg/m^2^)	27.97	±	5.28	26.20	±	3.45	30.30	±	3.74	25.15	±	3.48	36.87	±	6.04	< 0.0001
Neck	(cm)	40.27	±	3.74	39.36	±	3.05	41.73	±	2.96	38.59	±	3.18	45.07	±	3.95	< 0.0001
Waist	(cm)	95.50	±	13.29	90.25	±	9.58	99.73	±	9.72	89.51	±	10.44	115.22	±	13.90	< 0.0001
Hip	(cm)	104.17	±	11.03	101.81	±	7.40	108.68	±	7.66	98.30	±	8.69	123.41	±	10.93	< 0.0001
ESS score		9.65	±	4.90	14.40	±	3.15	8.40	±	3.45	5.63	±	2.60	13.70	±	4.03	< 0.0001

*ANOVA with Tukey with post hoc test, **Body Mass Index

*ESS* Epworth Sleepiness Scale

The cluster analysis identified four clusters. Compared with the Clusters 1 and 2, Cluster 3 was older and Cluster 4 youngest. There were no significant differences in the male-female ratio or history of smoking between the clusters. The BMI values, as well as the neck, waist, and hip circumferences, were the highest in Cluster 4. ESS scores were elevated in Clusters 1 and 4

Table 3 shows the PSG results. Of the enrolled OSA patients, 95 (9%) had mild (AHI 5–15/h), 200 (20%) had moderate (AHI 15–30/h), and 728 (71%) had severe (AHI > 30/h) OSA, with a mean AHI of 47(/h) and a mean oxygen desaturation index of 50/h. Sleep-disordered breathing data (obstructive apnea (OA), apnea index (AI), AHI, oxygen desaturation index 3%, mean SpO2, and arousal index) was the worst in Cluster 4, followed by Cluster 2.

**Table 3:** Sleep characteristics

		All clusters			Cluster 1			Cluster 2			Cluster 3			Cluster 4			*p* value
		*N* = 1023			*N* = 290			*N* = 271			*N* = 361			*N* = 101			
Awake	(min)	74.58	±	67.86	67.23	±	64.29	78.18	±	71.84	80.94	±	68.48	60.61	±	61.11	0.0162
REM	(min)	65.57	±	34.23	72.12	±	34.82	57.68	±	33.04	67.64	±	33.85	58.69	±	32.25	< 0.0001
Awake	(%)	14.64	±	13.53	12.91	±	12.36	16.01	±	15.22	15.67	±	13.37	11.78	±	11.69	0.0117
REM	(%)	12.80	±	8.54	14.37	±	12.34	11.41	±	6.30	12.94	±	6.37	11.19	±	6.35	0.0004
SPT		502.03	±	54.74	509.73	±	48.77	490.57	±	59.12	502.02	±	58.57	510.21	±	34.64	0.0004
TST		439.84	±	88.90	452.57	±	82.99	426.28	±	96.24	436.12	±	88.60	453.65	±	80.48	0.0040
SE		0.87	±	0.14	0.89	±	0.13	0.86	±	0.14	0.87	±	0.14	0.89	±	0.14	0.1526
CA	(%)	0.97	±	3.64	0.91	±	3.71	0.80	±	2.50	1.59	±	5.18	0.25	±	0.66	0.0118
OA	(%)	24.17	±	19.21	17.20	±	15.52	32.27	±	18.94	15.21	±	13.52	37.82	±	21.39	< 0.0001
MA	(%)	2.79	±	8.99	1.89	±	4.89	4.39	±	13.63	1.97	±	5.26	2.53	±	7.24	0.3506
AI	(/h)	30.52	±	25.02	21.38	±	18.45	49.23	±	22.39	18.29	±	15.52	59.27	±	28.61	< 0.0001
AHI	(/h)	47.43	±	24.98	36.17	±	18.66	69.63	±	15.19	31.74	±	15.37	76.30	±	21.55	< 0.0001
ODI3	(/h)	49.86	±	54.37	35.87	±	29.23	73.65	±	53.43	32.74	±	33.81	98.07	±	108.32	< 0.0001
Mean SaO2	(%)	93.37	±	3.95	94.63	±	2.51	91.78	±	3.55	95.04	±	1.97	88.01	±	6.52	< 0.0001
Arousal Index	(/h)	42.03	±	23.02	34.16	±	17.02	56.00	±	20.02	31.34	±	15.53	68.20	±	29.63	< 0.0001

*ANOVA with Tukey’s post hoc test

*REM*, rapid eye movement; *SPT*, sleep period time; *TST*, total sleep time; *SE*, sleep effect; *CA*, central apnea; *OA*, obstructive apnea; *MA*, mixed apnea; *AI*, apnea index; *AHI*, Apnea-Hypopnea Index; *ODI*, oxygen desaturation index

Of the enrolled OSA patients, 95 (9%) had mild (AHI 5–15/h), 200 (20%) had moderate (AHI 15–30/h), and 728 (71%) had severe (AHI > 30/h) OSA, with a mean AHI of 47/h and a mean oxygen desaturation index of 50/h. Sleep-disordered breathing data (obstructive apnea (OA), apnea index (AI), AHI, oxygen desaturation index 3%, mean SpO2, and arousal index) was the worst in Cluster 4, followed by Cluster 2

Table 4 displays the results of the lung function test. Although no ventilatory dysfunction was observed, the forced vital capacity (FVC) and the forced expiratory volume (FEV_1.0_) were the lowest in Cluster 4. There were no significant differences regarding the peripheral airway obstruction. The results of the arterial blood gas tests are shown in Table 4. Overall, CO2 tended to be high with metabolic compensation. Especially in Cluster 4, the mean value above 45 mmHg was substantially increased. Besides, oxygen at rest tended to be low in this cluster.

**Table 4:** Lung function tests and arterial blood gas analysis

		All clusters			Cluster 1			Cluster 2			Cluster 3			Cluster 4			*p* value
		*N* = 1023			*N* = 290			*N* = 271			*N* = 361			*N* = 101			
Lung function test																	
VC	(% predicted)	113.21	±	47.60	114.29	±	14.25	109.48	±	14.30	118.24	±	76.36	101.24	±	16.30	0.0094
FVC	(% predicted)	106.58	±	17.12	108.58	±	18.24	104.13	±	14.72	109.56	±	16.55	96.13	±	17.14	< 0.0001
FEV_1.0_	(% predicted)	100.19	±	16.59	103.10	±	15.95	97.16	±	13.62	103.73	±	16.98	86.55	±	15.92	< 0.0001
FEV_1.0_%	(%)	76.63	±	7.93	76.91	±	7.10	77.67	±	7.20	75.39	±	8.51	77.61	±	9.29	0.0035
MMF	(% predicted)	79.59	±	28.57	80.75	±	27.59	80.30	±	26.38	79.95	±	30.18	72.91	±	30.39	0.1249
V50	(% predicted)	69.98	±	24.11	71.39	±	24.55	71.90	±	22.62	68.38	±	23.88	66.67	±	27.06	0.1214
V25	(% predicted)	44.12	±	20.51	45.90	±	21.22	44.28	±	18.36	43.11	±	20.86	42.30	±	22.44	0.3016
Blood gas analysis																	
pH		7.40	±	0.03	7.40	±	0.02	7.40	±	0.04	7.41	±	0.03	7.39	±	0.02	< 0.0001
PaCO2	(mmHg)	43.43	±	4.07	43.95	±	3.41	42.96	±	3.29	42.12	±	3.71	47.88	±	5.45	< 0.0001
PaO2	(mmHg)	81.66	±	11.10	82.77	±	10.88	80.07	±	10.55	84.51	±	10.70	72.57	±	9.00	< 0.0001
HCO3	(mmoL/L)	26.59	±	1.92	26.72	±	1.71	26.38	±	1.71	26.21	±	1.88	28.23	±	2.23	< 0.0001
SaO2	(%)	96.17	±	1.57	96.38	±	1.45	95.86	±	1.63	96.57	±	1.34	94.86	±	1.71	< 0.0001

*ANOVA with Tukey’s post hoc test

*VC*, vital capacity; *FVC*, forced vital capacity; *FEV*, forced expiratory volume; *MMF*, maximal mi-expiratory flow

Although no ventilatory dysfunction was observed, the forced vital capacity (FVC) and the forced expiratory volume (FEV_1.0_) were the lowest in Cluster 4. There were no significant differences regarding the peripheral airway obstruction. Overall, CO2 tended to be high with metabolic compensation. Especially in Cluster 4, the mean value above 45 mmHg was substantially increased. Besides, oxygen at rest tended to be low in this cluster

According to the blood test data (Table 5), the white and red blood cell count, as well as the hemoglobin levels, were increased in Clusters 2 and 4. Similarly, liver function parameters, uric acid levels, and the values of a high-sensitivity test that detects C-reactive protein (CRP) were increased, particularly in Cluster 4.

**Table 5:** Blood chemistry analysis

		All clusters			Cluster 1			Cluster 2			Cluster 3			Cluster 4			*p* value
		*N* = 1023			*N* = 290			*N* = 271			*N* = 361			*N* = 101			
WBC	(×10^3^)	6.89	±	1.92	6.46	±	1.86	7.42	±	1.92	6.40	±	1.56	8.44	±	2.08	< 0.0001
RBC	(×10^6^)	4.76	±	0.49	4.73	±	0.47	4.80	±	0.46	4.66	±	0.49	5.11	±	0.50	< 0.0001
Hb	(g/dL)	14.91	±	1.44	14.87	±	1.27	15.00	±	1.50	14.66	±	1.48	15.65	±	1.35	< 0.0001
Hct	(%)	43.85	±	4.19	43.61	±	3.74	44.06	±	4.28	43.21	±	4.22	46.26	±	4.19	< 0.0001
Platelet	(×10^3^/μL)	250.09	±	60.15	245.45	±	55.68	251.72	±	58.47	251.25	±	63.92	254.46	±	62.55	0.4985
T-Bil	(mg/dL)	0.63	±	0.27	0.63	±	0.27	0.61	±	0.26	0.64	±	0.27	0.60	±	0.27	0.3994
AST	(U/L)	28.77	±	15.75	26.14	±	11.90	31.81	±	18.55	25.67	±	11.28	39.10	±	23.37	< 0.0001
ALT	(U/L)	39.64	±	33.03	34.58	±	26.63	49.03	±	40.66	30.32	±	21.19	62.34	±	43.37	< 0.0001
LDH	(U/L)	188.57	±	60.96	182.33	±	35.42	197.95	±	96.21	180.44	±	41.07	210.24	±	49.89	< 0.0001
ALP	(U/L)	235.83	±	68.98	224.55	±	60.98	243.17	±	71.25	235.07	±	70.69	250.51	±	73.50	0.0028
G-GTP	(U/L)	64.91	±	59.36	55.27	±	41.81	74.57	±	63.26	61.33	±	67.95	79.35	±	51.17	< 0.0001
T-Cho	(mg/dL)	208.64	±	38.54	206.34	±	35.98	210.87	±	36.91	205.94	±	41.26	218.69	±	38.11	0.0194
HDL	(mg/dL)	49.16	±	12.87	49.81	±	13.18	45.35	±	9.92	52.70	±	14.29	44.63	±	8.99	< 0.0001
LDL	(mg/dL)	122.24	±	34.38	121.01	±	32.21	123.82	±	33.96	119.53	±	35.51	131.56	±	35.84	0.0239
TG	(mg/dL)	187.79	±	129.52	178.58	±	115.30	214.40	±	159.88	170.25	±	118.04	206.58	±	102.11	0.0002
FBS	(mg/dL)	113.06	±	32.28	109.41	±	29.60	119.02	±	37.52	109.70	±	25.97	119.33	±	40.87	0.0014
HbA1c	(%)	5.69	±	0.93	5.59	±	0.79	5.76	±	0.88	5.54	±	0.91	6.24	±	1.16	0.0014
BUN	(mg/dL)	14.86	±	9.87	15.41	±	13.42	14.67	±	6.08	15.10	±	10.04	12.99	±	3.18	0.2174
Creatinine	(mg/dL)	0.88	±	0.84	0.91	±	0.85	0.94	±	1.14	0.84	±	0.68	0.80	±	0.14	0.3503
UA	(mg/dL)	6.20	±	1.46	6.07	±	1.45	6.57	±	1.36	5.87	±	1.41	6.83	±	1.54	< 0.0001
BNP	(pg/mL)	30.69	±	81.47	29.10	±	61.62	40.22	±	125.92	28.36	±	51.88	11.81	±	13.12	0.6852
IgE	(IU/mL)	226.05	±	311.73	40.00	±	9.64	164.00	±	211.39	128.38	±	129.56	568.40	±	477.03	0.0705
h-CRP	(ng/mL)	1616	±	3898	1472	±	3489	2029	±	1938	1121	±	1901	4786	±	12,132	< 0.0001

*ANOVA with Tukey’s post hoc test

*WBC*, white blood cells; *RBC*, red blood cells; *Hb*, hemoglobin; *Hct*, hematocrit; *T-Bil*, total bilirubin; *AST*, aspartate aminotransferase; *ALT*, alanine aminotransferase; *LDH*, lactate dehydrogenase; *ALP*, alkaline phosphatase; *G-GTP*, gamma-glutamyl transferase; *T-Cho*, total cholesterol; *HDL-C*, high-density lipoprotein cholesterol; *LDL-C*, low-density lipoprotein cholesterol; *TG TG*, triglyceride; *FBS*, fasting blood sugar; *HbA1c*, hemoglobin A1c; *BUN*, blood urea nitrogen; *UA*, uric acid; *BNP*, brain natriuretic peptide; *IgE*, immunoglobulin E; *h-CRP*, high sensitive C-reactive protein; *eGFR*, estimated glomerular filtration rate

The white and red blood cell count, as well as the hemoglobin levels, were increased in Clusters 2 and 4. Similarly, liver function parameters, uric acid levels, and the values of a high-sensitivity test that detect C-reactive protein (CRP) were increased, particularly in Cluster 4

### Cluster analysis

#### Cluster 1 (290 subjects, 28.3%); middle age, symptomatic severe OSA

This cluster was centered on the middle-aged participants with a mean age of 51.00 ± 12.71 years. The mean BMI of patients belonging to this cluster was 26.20 ± 3.45(kg/m^2^). Based on the BMI (kg/m^2^) classification (normal weight, 18.5–24.9; overweight, 25.0–29.9; and obese > 30), these patients were mildly overweight. The mean ESS score of this cluster was 14.40 ± 3.15, indicating the most symptomatic excessive daytime sleepiness (EDS) of all clusters. The AHI, however, was with 36.17 ± 18.66(/h), far below the overall average.

#### Cluster 2 (271 subjects, 26.5%); middle age, obese, minimally symptomatic, very severe OSA

Like Cluster 1, this cluster was centered on the middle-aged participants with a mean age of 49.10 ± 12.71 years. Their mean BMI was 30.30 ± 3.74(kg/m^2^), with many patients found to be obese. The mean ESS score of this cluster was 8.40 ± 3.45, making this cluster a population with a low ESS score and a decreased likelihood to present with EDS symptoms among all clusters (Table 2). Yet, the AHI was 56.00 ± 20.02(/h), representing severe apnea and hypopnea.

#### Cluster 3 (361 subjects, 35.3%); oldest age, obese, minimally symptomatic, severe OSA

This cluster was centered on middle-aged patients with a mean age of 55.24 ± 13.29 years. The mean BMI of patients belonging to this cluster was 25.15 ± 3.48(kg/m^2^), indicating that they were mildly overweight. The mean ESS score of this cluster was 5.63 ± 2.60, suggesting the most limited EDS symptoms of all clusters (Table 2). The AHI was 31.34 ± 15.53(/h), which was far below the overall mean for AHI. This cluster was accordingly composed of non-obese OSA patients, equivalent to mild-to-moderate symptoms (Table 3).

#### Cluster 4 (101 subjects, 9.9%); young age, obese, symptomatic very severe OSA

This cluster was composed of relatively young patients with a mean age of 42.71 ± 10.96 years. The mean BMI of these patients was 36.87 ± 6.04(kg/m^2^), indicating severe obesity. The mean ESS score of this cluster was 13.70 ± 4.03, suggesting the strongest EDS symptoms of all clusters (Table 2). Accordingly, with an AHI of 68.20 ± 18.66(/h), this cluster presented the most severe OSA of all clusters (Table 3). Respiratory function testing with a FEV_1.0_% of 77.61% did not detect airflow obstructions, but the FEV_1.0_ of 86.55% was significantly lower than that in other clusters (Table 4). As shown by the blood gas analysis, PaO_2_ at 72.57 ± 9.00 Torr was the lowest despite this group being the youngest of all cluster populations. The PaCO2 was 47.88 ± 5.43 Torr indicating hypercapnia (Table 5). Blood tests also showed higher levels than other clusters with the white blood count at 8.44 ± 2.08/μL and h-CRP at 4.8 ± 12 μg/mL. GOT and GPT indicated hepatic dysfunction with 39.10 ± 23.37 and 62.34 ± 43.37 U/L, respectively. T-Cho, LDL, HbA1c, and UA also exhibited the highest values out of all clusters (Table 6).

**Table 6:** Adipokines

		All clusters			Cluster 1			Cluster 2			Cluster 3			Cluster 4			*p* value
		*N* = 167			*N* = 68			*N* = 31			*N* = 55			*N* = 13			
Leptin	(ng/mL)	8.45	±	6.91	7.11	±	5.47	12.15	±	6.69	5.77	±	4.98	18.01	±	9.65	< 0.0001
Adiponectin	(μg/mL)	4.24	±	4.57	3.73	±	2.57	4.72	±	6.11	4.96	±	5.76	2.77	±	1.58	0.2746
Leptin/adiponectin	3.55	±	4.87	2.95	±	3.94	4.54	±	3.29	2.08	±	2.87	10.59	±	10.44	0.0013	

*ANOVA with Tukey’s post hoc test

Serum leptin and adiponectin levels were determined in 167 patients whose samples were still available and compared between the four clusters (Table 6). Of the identified clusters, Clusters 1 and 4 exhibited high leptin levels. Adiponectin/leptin ratios and adiponectin levels showed no clear differences between clusters

#### Comparison of serum leptin and adiponectin levels

Serum leptin and adiponectin levels were determined in 167 patients whose samples were still available and compared between the four clusters (Table 6). Of the identified clusters, Clusters 1 and 4 exhibited high leptin levels. Adiponectin/leptin ratios and adiponectin levels showed no clear differences between the clusters.

## Discussion

### Summary of result

Four common factors (ESS, BMI, AHI, and PaCO2) were extracted by principal component analysis, and the cluster analysis revealed four clusters of various phenotypes with different clinical features. The subjects were middle-aged patients around 50 years old, mostly male, tended to be obese, but did not tend to have high daytime sleepiness. As a result, severe OSA was more frequent.

Our research was inspired by previous studies, and we considered that phenotyping could influence the actual clinical practice. The anticipated result was that items that have been previously linked to the severity of OSA, such as age [13], obesity [14], and daytime sleepiness [15], would be extracted as principal components. Previous reports made it clear that it is important to distinguish OSA types by phenotype [16]. However, these studies were not able to explain how phenotype and endotype are linked to this disease. We suggest that in all cases, the arterial blood gas analysis and leptin measurements are important tests to clarify the impact of OSA on respiratory control and lipid metabolism, i.e., the relationships between phenotypes and endotypes.

### Distinguishing factors of the identified subpopulations

Interestingly, no significant differences between men and women were observed for each cluster. Cluster 4 had a significantly higher rate of smokers (37% smokers; 61% if ex-smokers were included). Cluster 1 (middle age, symptomatic severe OSA) and Cluster 4 (young, obese, symptomatic very severe OSA) exhibited high leptin levels. C-reactive protein levels were also elevated in Cluster 4. In this group, the CRP and leptin levels were especially high indicating a different pathophysiological background, such as systemic inflammation or metabolic disorder, which we believe increases the cardiovascular risk [17]. Although not significantly different, Cluster 4 did exhibit a notably abnormal glucose tolerance. Counterregulatory hormones of insulin and the sympathetic nervous system impair the glucose utilization in brain tissue, reduce insulin secretion in pancreatic β cells, and decrease insulin sensitivity in peripheral tissues, thereby leading to abnormal glucose metabolism [18].

### Shortcomings of ESS and AHI in defining these subgroups

The correlation of the ESS score to indices of sleep-disordered breathing has been reported [12], but the classification into phenotypes generated groups with no correlation between the ESS score and severity of OSA. This suggests that sleepiness is affected by multiple elements, such as the duration of sleep, arousal response, and hypoxemia, which are not well reflected by this medical questionnaire.

Our data also show that severe OSA patients could not be described by AHI alone. This is because it is not possible to distinguish a disease state such as obesity hypoventilation syndrome (OHS). OHS is defined as hypoventilation (PaCO2 > 45 mmHg) and obesity (BMI > 30), after the exclusion of other causes. The direct measurement of CO2 levels is often difficult; nevertheless, the assessment of ventilation abnormalities, as performed in the present study, is essential for the determination of OSA severity. There are also differences in the metabolic dysfunction in each cluster so that it is difficult to determine the disease prognosis by AHI alone neither.

### Subgroup-specific links to adipokines

Obesity is a major factor for developing OSA; therefore, adipokine is critical in OSA pathophysiology. Most importantly, adipokine-leptin is closely related not only to obesity but also to the ventilation response. As a result, OSA patients have an increased leptin resistance as noted in past reports [19]. Leptin, however, was not a sufficient biomarker. This study classified the patients into characteristics and compared the leptin levels. We believe that leptin may be useful as a biomarker for OSA if such phenotyping is used.

### Consequences for clinical practice

To improve patient’s outcome, physicians would want to identify patients who would respond best to continuous positive airway pressure (CPAP) therapy and the patients that are at most risk for cardiovascular disease. In our study, we hypothesized that the effects of CPAP were high in groups that had metabolic dysfunction or central nervous system resistance to leptin’s ventilation promoting effect. We also hypothesized that these groups could have reduced cardiovascular risk [20–22].

In this context, the extent to which CPAP therapy ensures a sufficient sleep duration should be taken into consideration. A short sleep duration results in lower leptin and elevated ghrelin levels, increasing the appetite and, thus, causing a vicious cycle with the progressing obesity [23].

A limitation of this study is that it is a retrospective study and that the sample size is small. Also, we did not consider the daily sleep duration or complications such as hypertension, cardiovascular disease, and diabetes. Prospective studies regarding the effects of CPAP therapy are needed.

## Conclusion

The existence of multiple clinical phenotypes suggests that different pathophysiological backgrounds exist, such as systemic inflammation and metabolic disorder. This classification may be used to predict the efficacy of continuous positive airway pressure treatment that cannot be determined by the AHI.

## Data Availability

All data generated or analyzed during this study are included in this published article.
